# Achievements and Challenges upon the Implementation of a Program for National Control of Congenital Chagas in Bolivia: Results 2004–2009

**DOI:** 10.1371/journal.pntd.0002304

**Published:** 2013-07-11

**Authors:** Cristina Alonso-Vega, Claire Billot, Faustino Torrico

**Affiliations:** 1 APEFE, Programa Nacional de Control de Chagas, Ministry of Health, La Paz, Bolivia; 2 Facultad de Medicina, Universidad Mayor de San Simón (U.M.S.S.), Cochabamba, Bolivia; National Institutes of Health, United States of America

## Abstract

Bolivia is one of the most endemic countries for Chagas disease. Data of 2005 shows that incidence is around 1.09‰ inhabitants and seroprevalence in children under 15 ranged from 10% in urban areas to 40% in rural areas. In this article, we report results obtained during the implementation of the congenital Chagas program, one of the biggest casuistry in congenital Chagas disease, led by National Program of Chagas and Belgian cooperation from 2004 to 2009. The program strategy was based on serological results during pregnancy and on the follow up of children born from positive mothers until one year old; if positive, treatment was done with Benznidazole, 10 mg/Kg/day/30 days with one post treatment control 6 months later. Throughout the length of the program, a total of 318,479 pregnant women were screened and 23.31% were detected positive. 42,538 children born from positive mothers were analyzed at birth by micromethod, of which 1.43% read positive. 10,120 children returned for their second micromethod control of which 2.29% read positive, 7,650 children returned for the serological control, of which 3.32% turned out positive. From the 1,093 positive children, 70% completed the 30 day-treatment and 122 returned for post treatment control with 96% showing a negative result. It has been seen that maternal-fetal transmission rates vary between 2% and 4%, with an average of 2.6% (about half of previously reported studies that reached 5%). In this work, we show that it is possible to implement, with limited resources, a National Congenital Chagas Program and to integrate it into the Bolivian health system. Keys of success are population awareness, health personnel motivation, and political commitment at all levels.

## Author Summary

Congenital Chagas disease is the infection resulting of the transmission of *T.cruzi* parasites from an infected pregnant woman to her fetus during the pregnancy or at the time of delivery. This represents a relevant public health issue in endemic and non-endemic countries. The infected newborn detected at birth or before one year old, if treated, can be completely cured. In this work, we report results obtained from 2004 to 2009 during the implementation of a National Program based on PAHO recommendations; the implementation strategy has been adapted into the Bolivian context and integrated into the National Mother and Child Health program. Our conclusions show that it is necessary to test all newborns from positive mothers three times before one year of age. The infant, if positive and treated with a 30 day Benznidazole course, has excellent chances of cure.

## Introduction

Chagas' disease or American trypanosomiasis, is an antropozoonosis caused by the protozoan *Trypanosoma cruzi* – a blood and tissue parasite. It currently affects 15 million people, produces more than 15 000 deaths each year and is the most socially and economically impacting parasitic disease in the Americas [1] [2].

The infection can be contracted by vector transmission – through the feces of haemophagic vectors of the reduvidae family and the triatominae sub-family, via blood transfusion or organ transplants, via vertical or congenital transmission from an infected mother to her new born child or fetus and by oral transmission. Other transmission mechanisms, with a minor epidemiological importance, include organ transplantation or laboratory accidents.

The progressing vector control, along with the migration tendencies within and from endemic countries, has modified the distribution of the Chagas disease within the last years, and it has augmented the relative importance of the congenital transmission route. According to a report from the Chagas scientific work team in 2005, the annual incidence of congenital Chagas would be of over 14,000 newborns. The disease is now present and has become a public health concern in the whole American continent, Europe, Japan and Australia [3], [4], [5], [6], [7].

The congenital transmission of Chagas disease was documented for the first time in 1911, by Carlos Chagas, who had found parasites in the necropsies of two twins with convulsion episodes that died a few days after birth. Later, Dao in Venezuela (1949), Jorg in Argentina (1953), Howard in Chile (1957) and Bittencout and Rezende in Brazil (1959), all describe the first cases in their respective countries [8]. Chagas congenital disease has also been reported in other endemic countries such as Uruguay, Paraguay, Colombia, Guatemala, Honduras and Mexico. Since then numerous articles have been published regarding congenital Chagas disease: epidemiological and clinical studies, evaluation of different diagnostic methods [9], congenital Chagas as an imported disease (cases reported in Spain, USA, Switzerland), and above all, highlighting the importance of the disease in terms of public health and the need for health programs concerned with its diagnosis and treatment in all the endemic countries [7], [10], [11], [12], [13], [14], [15]. However, the initiatives for control and management of congenital Chagas in endemic countries are far from achieving total geographic coverage [16], [17], [18], [19], [20], [21], [22]. Such initiatives are not only necessary in endemic countries, but should, in a targeted way, also be implemented in countries that receive or have received significant migration flows from Latin America such as USA, Spain, Switzerland and others [3], [23], [24].

The first congenital record in Bolivia is accredited to Azogue and col. in 1981. They describe an 8% transmission rate in the “Percy Boland” maternity unit of Santa Cruz de la Sierra [25]. The same authors have published various articles on studies carried out in the 80s, in which they highlight the importance of detecting congenital Chagas in areas where the vector is controlled. Also they recommend the treatment of women in fertile age, they found hepatosplenomegaly as the most common sign in newborns and used the Strout technique in cord blood as a sensitive and less expensive diagnosis technique [26], [27].

Between 1991 and 1994, the Chagas control program of the National Health Department of Bolivia detected a maternal seroprevalence of 27.6% and a maternal-fetal transmission rate of 4.9% [28], [29].

In 1998, the Free University of Brussels (ULB) (Brussels – Belgium) and the IIBISMED of the faculty of Medicine of the UMSS (State University of Cochabamba) began a joint research project on mechanisms involved in congenital transmission of Chagas. The transmission rate in this study was 5%, and it was our reference at the beginning of the congenital Chagas program [28]. The results of this project, along with the investigations of international experts on congenital Chagas, were discussed in a conference which took place in Cochabamba, 2002 [30], [31]. After that, the PAHO organized a technical meeting in Montevideo (Uruguay, 2004), where a sustainable and effective strategy for detection and treatment of congenital Chagas was designed. This strategy is currently still in effect and its main directive is: “To carry out intervention and control activities to prevent and control congenital infection by *Trypanosoma cruzi*, due to the importance that the latter has on children's health and the epidemiology of the parasitosis”, implying that such control measures have not yet been implemented in endemic countries [32].

In this article, we report results attained from 2004 to 2009 during the implementation of the congenital Chagas program in Bolivia, and present recommendations of the course to follow in order to maintain and improve this program.

## Methods

### Detection strategy of congenital Chagas disease

The congenital Chagas component of the National Chagas program in Bolivia started in 2004 based on the recommendations of the PAHO [32]. The activities revolve around three axes of action: health personnel training, laboratory diagnosis and IEC.

#### Diagnosis of infection in pregnant women

The detection of infection in pregnant women was achieved by prenatal serological screening. If at the moment of delivery the woman did not have a result on the test or had not been studied, an intravenous blood sample was taken pre-partum, postpartum or the sample was taken from the cord blood in order to perform Chagas serology.

The serology of Chagas was carried out with the Indirect Hemaglutination (IHA) and/or ELISA technique, using commercial kits (HAI Chagas – Polychaco-Buenos Aires, Argentina, ELISA Bios Chile, ELISA Wiener, Argentina).

In practice, few public health centers had access to an ELISA reader. For this reason the IHA technique was employed in most centers for screening pregnant women, using the 1/16 dilution measure as the cut-off line [17] [33].

The serological test results were noted in the women's clinical history and prenatal ID card; women with positive results had to be followed up for the monitoring of their child until 12 months of age.

#### Monitoring children born from a Chagas' positive mother

If the woman was positive at the moment of birth, a blood sample was taken from the umbilical cord in a heparinized tube. Alternatively, a peripheral blood sample was taken from the newborn in 4 heparinized capillary tubes.

This blood sample was taken to the laboratory and immediately tested using the micromethod technique as described in the literature, [33], [34], [35], [36]. Samples had to be less than 12 hours old; samples that were over 12 hours old were discarded and a new peripheral blood sample was taken from the child in heparinized capillary tubes. This analysis was carried out by personnel who was trained in the detection of *T.cruzi*.

In case of a positive micromethod, the result was to be immediately communicated to the doctor or pediatrician on duty and treatment of the newborn was started before the mother and child left the maternity unit.

If the micromethod was negative at the time of birth, another micromethod was carried out normally between 15 days and 2 months of delivery but definitely before 6 months.

**Figure 1:**
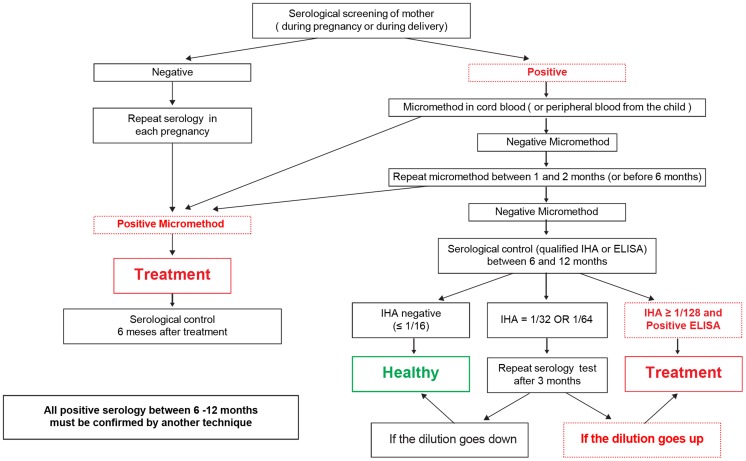
Diagnostic Diagram of congenital Chagas disease in newborns.

Starting from 6 months and up to 12 months of age, if both micromethods were negative, a quantified serology was carried out on the child. The IHA method was employed due to its ease of use and accessibility at most of the laboratories. If the serology was negative, the child was discharged; if it was strongly positive at a dilution higher or equal to 1/128, the result was confirmed with another test (generally ELISA) and the child was considered positive and treatment started. If the IHA was a dilution between 1/16 and 1/64, the quantified serology was repeated within the 12 months of age (or more if necessary) and was evaluated according to the manual [33]. (Figure 1). In practice, the cases of an inconclusive serology generally happened between 6 and 8 months, starting from 8 months onward, the serology usually turns out negative or strongly positive (data not show).

### Treatment and follow up of children with congenital Chagas

All children diagnosed with congenital Chagas were treated with Benznidazole; 10 mg/kg/day for 30 days, in two doses – complying with the current regulations in Bolivia. The first week was begun with 7 mg/kg/day in two doses. Considering that Benznidazole does not yet come in pediatric form, the medicine was dosed in the following manner: a tablet of 100 mg of Benznidazole was mixed into 10 milliliters of drinking water, producing a suspension of 10 mg/ml (being a liposoluble medication, it cannot be diluted in water, but with a vigorous stir, a suspension is produced). Dosages were calculated according to the weight of the patient and were administered with the aid of a graduated syringe in order to improve the precision of quantity required. Children undergoing treatment were monitored on a weekly basis in order to adjust the dosage according to their weight, evaluate the compliance with treatment and rule out any possible adverse effects [37].

In order to avoid a second infection through the vector, it was advised that a vector control technician should visit the home address of the child undergoing treatment and spray the house if considered necessary.

Six months after treatment was finalized, a serological test was performed to the child in order to certify the cure. In case of persistent positive serology, the test was repeated 6 months after and if still positive, the initial treatment ruled as “failed” and the treatment was repeated.

### Integration into the Bolivian health system

In order to implement the strategy for congenital Chagas at a health facility, an evaluation of the conditions had to be carried out beforehand, particularly regarding the laboratory. The minimum equipment required was a microscope with a 40× lens, a hematocrit micro-centrifuge, a macro centrifuge, and adjustable-volume pipettes. Also, a reference laboratory with an ELISA reader is needed.

The integration of the congenital Chagas program with the country health system has been gradual and steady, and with the financial support of the French Community of Belgium cooperation project (WBI). At the beginning there were ten facilities supporting the departments of Cochabamba (3), Tarija (4) and Chuquisaca (3).

The integration into the facility's routines started with an initial training of the health personnel and individuals in charge of gynecology, pediatrics, laboratory, and nursing sections. It was essential to form a solid work team, since the coordination between different sections was crucial for running the program. This initial training consisted of a theoretical part: The Chagas disease, diagnosis and treatment of Chagas and congenital Chagas followed by a practical part: laboratory procedures using the IHA, ELISA and micromethod techniques (using 3T3 cells cultivated with *T.cruzi*) [38]. Once the activities had begun, part of the health personnel was trained on using IEC components. Between three to six months later, the first supervision visits took place. These visits were made once or twice a year but more often if problems were detected at a particular facility.

Monitoring indicators were designed and established in order to strengthen the program's integration into the health facilities. These allowed the facilities to grade their coverage according to their level of service. (Table 1)

**Table 1:** Description of the indicators used in the congenital Chagas program.

Concept	Source of data	Indicator	Target	Type of health facility
a. - Serology coverage in pregnant women.	**Laboratory:** n° of serology tests carried out in CPN#, **SNIS** ****: n° of new CPN attended at the facility	N° of serology tests performed in CPN/N° of new CPNs in the facility ×100	90%	All
b. - Coverage of micromethods performed at birth on children born from positive mothers.	**Laboratory:** n° of micromethods performed at birth, maternal seroprevalence. **SNIS**: n° of births and caesareans attended at the facility	N° of Micromethods performed at birth/n° of births expected from positive mothers ×100**	75%	Only facilities that have maternity units
c. - Coverage of control between 15 days and 12 months of age.	**Laboratory:** n° of micromethods performed between 15 days and 6 months of age in children born from positive mothers.	N° of micromethods performed between 15 days and 6 months of age/n° of expected births from positive mothers ×100	50%	Level 1 and 2 facilities
d.- Coverage of control between 6 and 12 months of age	**Laboratory:** n° of serology tests performed between 6 and 12 months of age.	N° of serology tests performed between 6 and 12 months/n° of births expected from positive mothers* ×100	50%	Level 1 and 2 facilities
e.- Detected cases	**Laboratory:** n° of detected cases	N° of detected cases/n° of expected cases ×100***	50%	All
f.- Completed treatment	**Clinic History:** cases of treatment completed, **Laboratory:** number of detected cases	N° of treated cases/n° of detected cases ×100	80%	All
g.- Information sent out to SNIS	**SNIS**		100%	All
h. - costs covered by Universal Mother-child Insurance.	**Service administration**		100%	All

# CPN: Pre natal control.

** n° of births expected from positive mothers = n° of births and caesareans attended×seroprevalence in pregnant women (%).

*** n° of expected cases = n° of births expected from positive mothers ×3% (transmission rate).

**** SNIS: National Health Information System.

During the development of the program, Chagas indicators were introduced into the national epidemiologic health information records. In 2008, modifications were introduced into the monthly and weekly record forms so that the information recorded would reflect the country's epidemiological reality and the activities that were being carried out in the fight against the disease. The forms are available online in the Health and Sports Ministry website (http://www.sns.gob.bo/snis/default.aspx). In 2009, following the recommendations made at the meeting held in Montevideo in 2004 regarding the inclusion of basic congenital Chagas details within the Perinatal Information System or CLAP sheet [39], the variables concerning diagnoses of Chagas in pregnant women and newborns were introduced into the Bolivian version of the CLAP sheet and into the child heath ID cards, in order to ensure the flow and availability of information. These modifications in the current forms have been supported by the ministerial resolution N° 1321 of 28/12/2009.

Towards the end of 2009 the goal of the program was almost achieved, with most facilities complying by sending monthly reports regarding congenital Chagas, in coordination with departmental National Health Information System (SNIS) personnel.

Since 2004, the laboratories included in the congenital Chagas program have gone through successive quality control tests in Chagas serodiagnosis, by means of serum panels and/or sample retesting. Between 2004 and 2006, quality control was the responsibility of the University staff and the project staff. Since 2007, this responsibility was delegated to the departmental reference laboratories. The national reference laboratory for Chagas CENETROP, in turn, performed quality checks on the departmental reference laboratories in 2008 and 2009.

## Results

### Diagnosis and treatment of Chagas congenital disease

From June 2004 to December 2009 the program was implemented in 90 health facilities in 49 out of the 168 municipalities located in endemic areas (Andean mesothermic valleys, tropical zones, the Chaco dry forest, and the Altiplano). As a result, 29% of all municipalities within endemic areas were covered.

The 90 health facilities are comprised of 7 urban 3^rd^ level hospitals, 35 2^nd^ level hospitals (5 located in departmental capitals and 30 in province areas), and 48 1^th^ level facilities (35 located in the capitals and 13 in provinces). These 90 facilities represent 73% of all health facilities with laboratory in the selected municipalities considering a denominator of 123 facilities (data from Ministry of Health, Bolivia, http://www.sns.gob.bo/).

**Figure 2:**
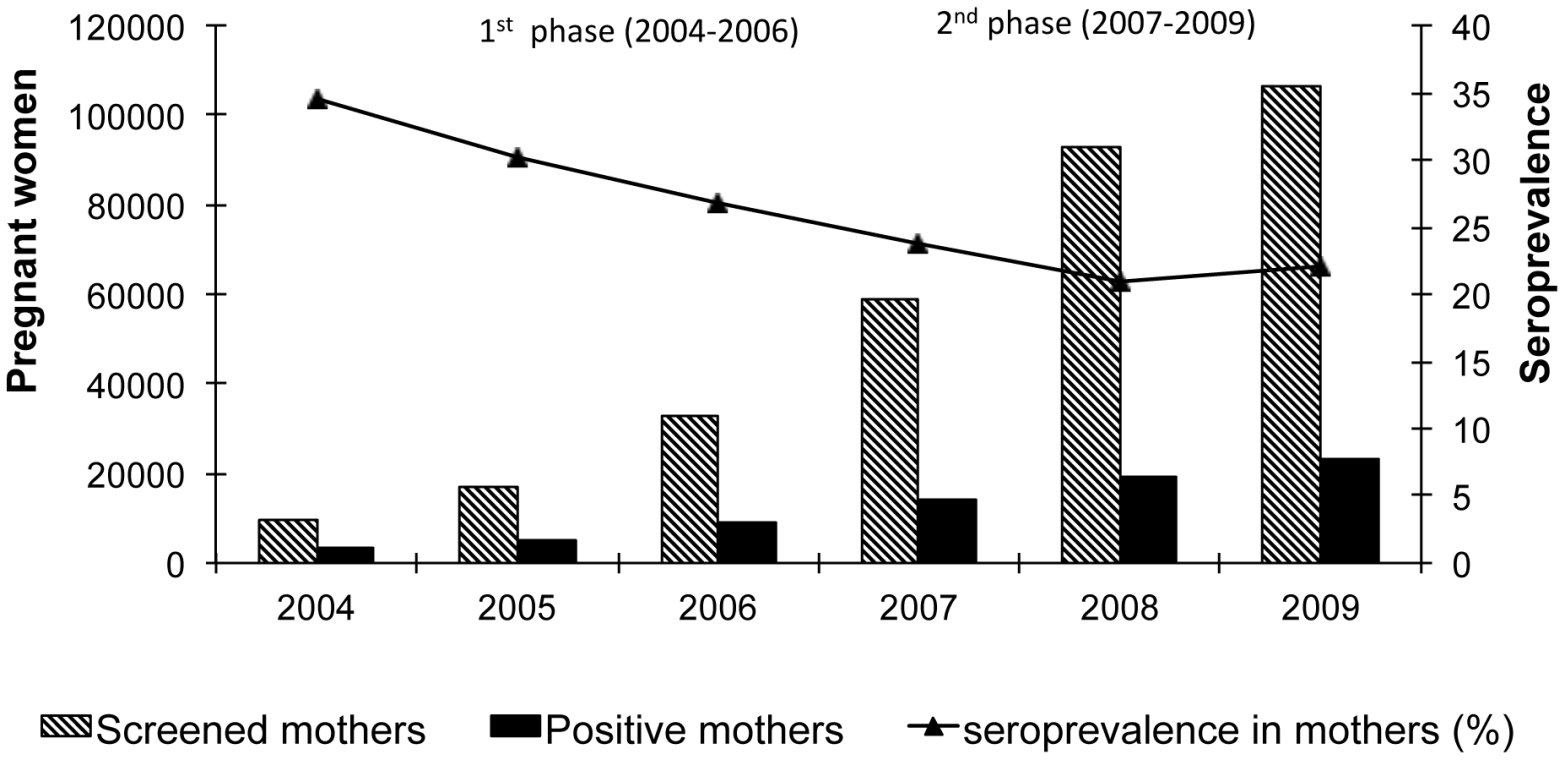
Total of pregnant women screened and seroprevalence 2004–2009. The 1^st^ phase includes the departments of Cochabamba, Tarija, and Chuquisaca, with a large seroprevalence in Chagas disease. The 2^nd^ phase additionally includes the departments of Santa Cruz, La Paz and Potosi, with a lower seroprevalence. The average prevalence decreases as the total of women screened increases in less endemic areas.

From the beginning of the project in 2004 until December 2009, a total of 318,479 pregnant women were screened for Chagas and 74,228 were detected positive (23.3%). Considering that the project was comprised of 2 phases: the first from 2004 to 2006, consisting of implementations in health facilities of Cochabamba, Tarija and Chuquisaca, and the second, in 2007 to 2009, consisting of the expansion into the departments of Santa Cruz, La Paz and Potosi, we can appreciate an important increase in the number of women screened since 2007. The average maternal seroprevalence shows a decrease with time as the program extends into more areas and it stabilizes around 22% since 2008 and after (Figure 2).

Table 2 shows that, during 2009, more than 100% of pregnant women registered in each of the 90 health facilities were screened (source: data from SNIS and from Chagas congenital program). This data indicates bias in the prenatal screening and is due to repeated tests; i.e. same women tested in different facilities. This is one of the operational problems in the Bolivian health system that could not be solved.

**Table 2:** Pregnant women screened compared with pregnant women registered in health facilities and seroprevalence (2009).

Department	Chuquisaca	Tarija	Cochabamba	Santa Cruz	La Paz	Potosí	TOTAL
N° of women screened	11,568	9,325	39,071	38,830	2,520	5,410	**106,724**
Pregnant women registered	7,401	6,762	36,612	27,751	1,016	3,666	**83,208**
Seroprevalence (Confidence interval)	37% (36–38)	38% (37–39)	18% (17–18)	20% (19–20)	5% (4–6)	9.5%(9–10)	**22% (21–22)**

In the 6 endemic departments of Bolivia, the number of women tested is greater than the number of women registered in each hospital. This fact is due to numerous failures in clinical records that lead to repeat testing in prenatal controls.

Between 2004 and 2009, a total of 42,538 children born from positive mothers were analyzed at birth using the micromethod, 606 (1.4%) of which read positive. Only 10,120 (24%) returned for their second micromethod control, and 232 (2.3%) of these read positive. For the serological control between 6 and 12 months, 7,650 (18%) returned, with 254 (3.3%) turning out positive. A number of 24,767 newborns from positive mother did not do the follow-up for multiples reasons such as failures in the reference system, failures in the communication with the mother, low health education levels, geographical barriers in the accessibility to the facilities, etc.

**Figure 3:**
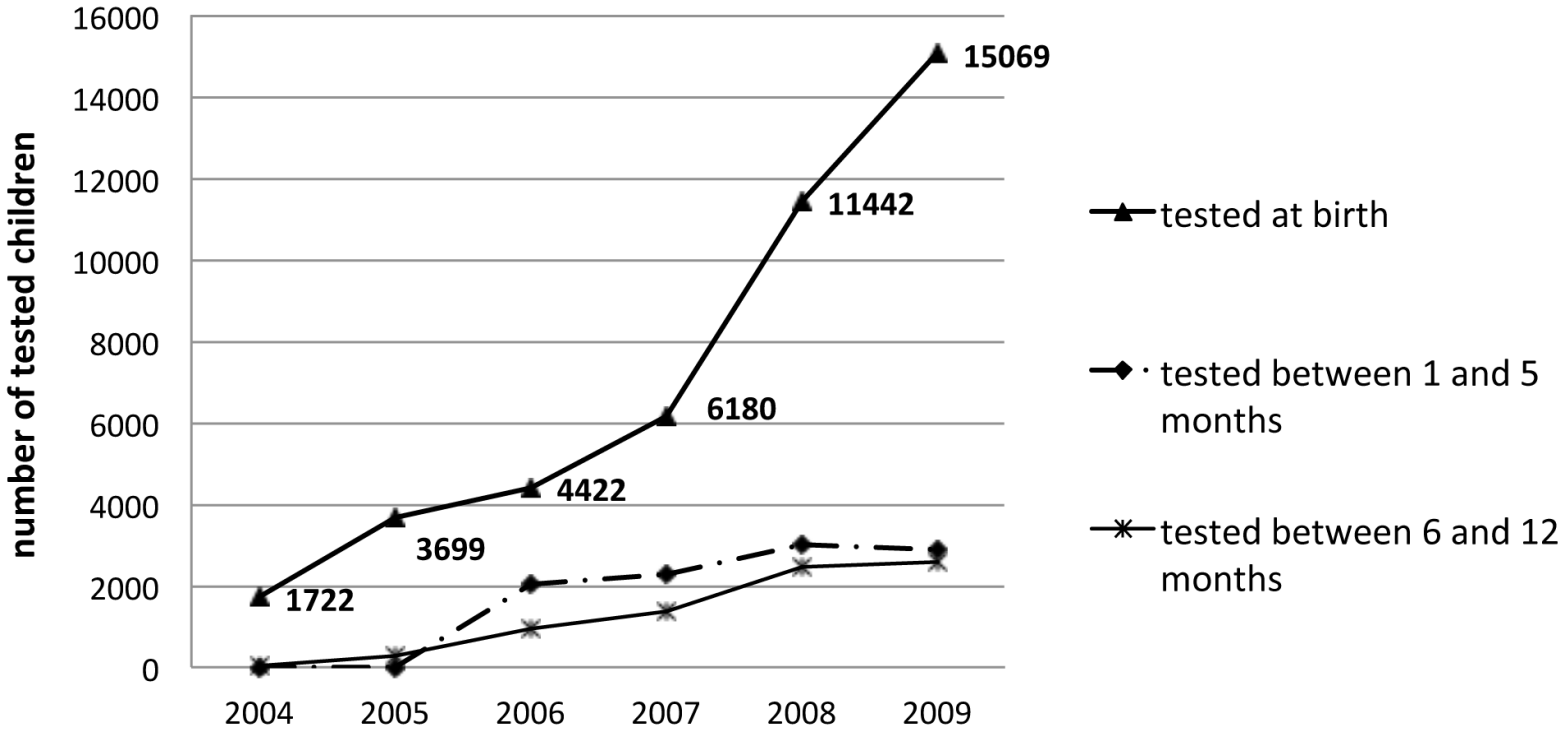
Follow up of children born from positive mothers between 2004 and 2009. Children tested at birth increased due to the inclusion of big maternities in the program; however, the number of children followed after birth, did not increase proportionally.

In Figure 3 we can see that there is an increasing number of children controlled at birth according to the number of facilities participating in the program, which reflects a higher number of mothers screened. However, we cannot see a proportional increase in tests performed after birth.

**Figure 4:**
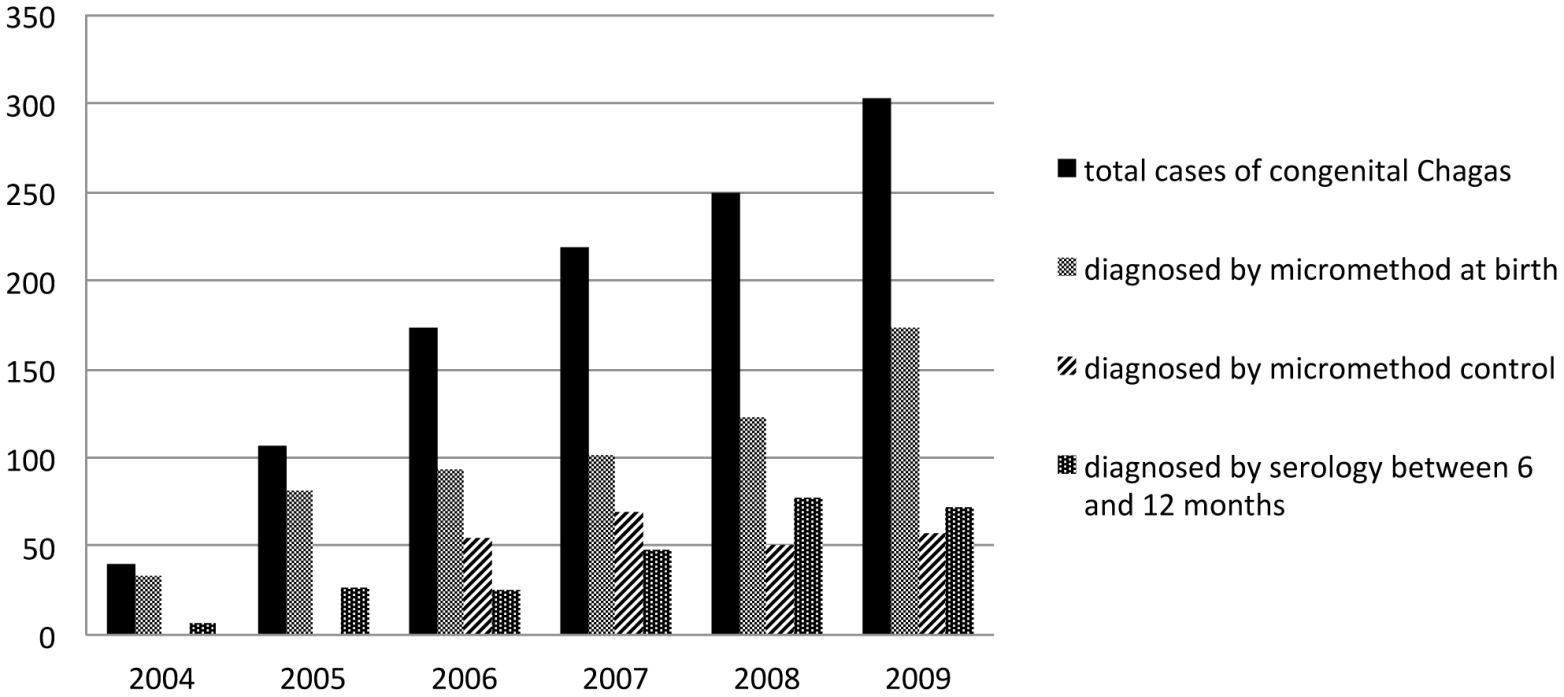
Cases diagnosed according to the method used and time of follow up. **2004–2009.** Most of the congenital cases were diagnosed by micromethod before six months of age (micromethod at birth and micromethod control).

There were a total of 1,093 congenital Chagas diagnosed cases. As seen in Figure 4, the number of cases diagnosed has continually increased as the number of health facilities entering the program has gone up, from 40 cases in 2004 (10 facilities) to 303 cases in 2009 (90 facilities). If we calculate 5% of 42,538 newborns of positive mothers studied at birth (5% was our reference for congenital transmission before starting the program [28]), 51% of expected cases have been detected (1,093/2,127).

From the 1,093 cases diagnosed, 55% have been diagnosed at birth; a logical consequence considering the greater number of tests carried out at that time compared to the number of tests done after birth. The rest of the children have been diagnosed in relative proportion to the following tests, 21% in parasitological testing before 6 months and 24% in serological testing between 6 and 12 months.

**Figure 5:**
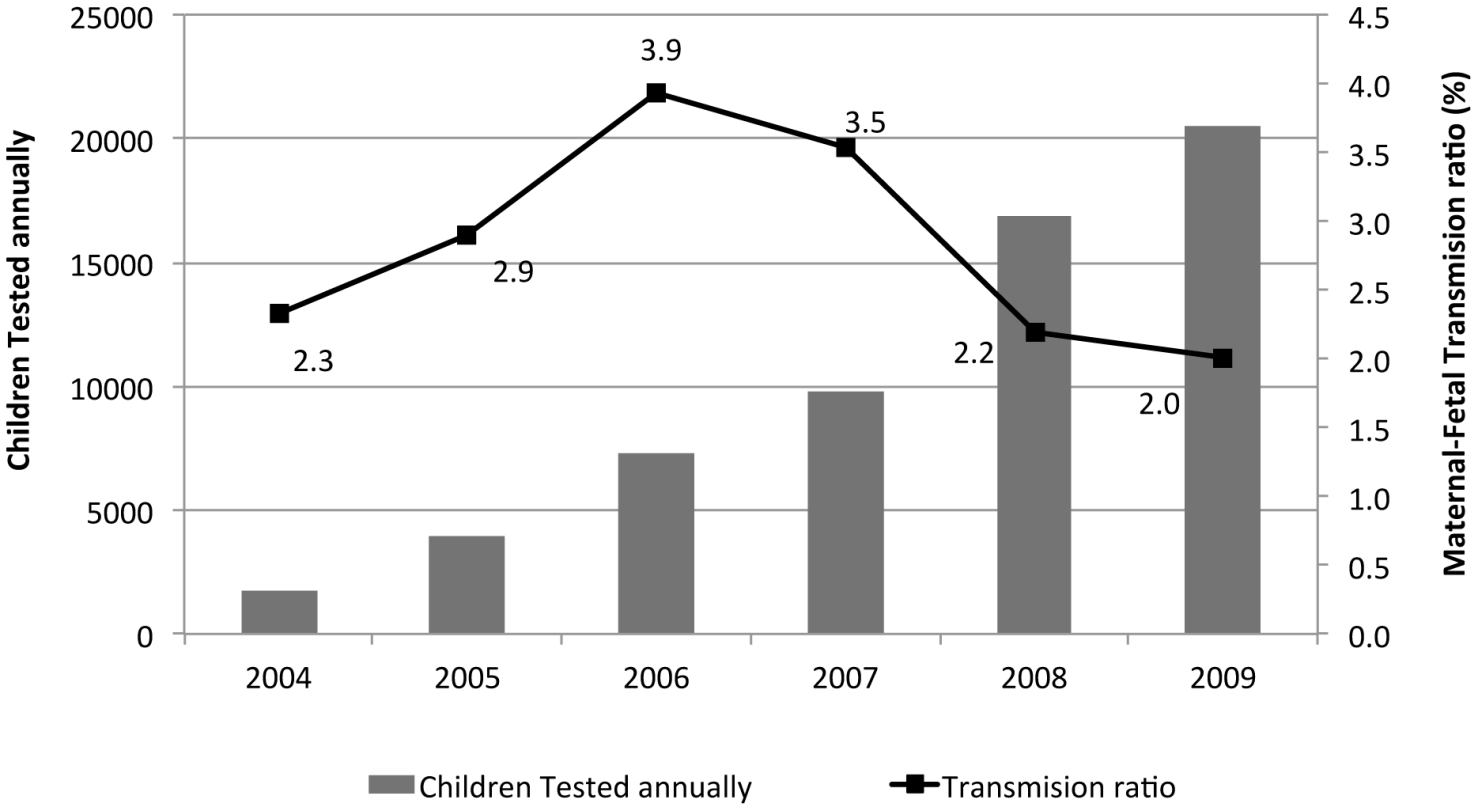
Maternal - Fetal transmission ratio and controls carried out on children less than one year of age born from positive mothers. The Maternal-Fetal transmission ratio is calculated with the total number of congenital Chagas cases per year, over the number of children born from positive mothers tested at birth. The ratio decreases as the number of annually tested children increases.

The maternal-fetal annual transmission rate varies between 2% and 4%, with an average of 2.6%, which is below the expected 5% (Figure 5). Those rates were calculated with the total cases diagnosed per year over the total newborns from positive mothers controlled at birth. No correlation has been seen between the total number of tests carried out and the detected transmission rate, however, we can observe that in the last two years the maternal-fetal annual transmission rate has stabilized at 2%.

In Tables 3 through 8, data is displayed by health facility/network from 2004 to 2009. We can see that the seroprevalence found in pregnant women does not go through significant changes along the years of implementation in the various intervention areas. The variations in seroprevalence found were due only to the increase in health facilities within such geographical areas, with different epidemiological characteristics; and, above all, related to previous infestations rates [40]. Therefore, the seroprevalence in women is not related with transmission rates.

**Table 3:** Seroprevalence and maternal-fetal transmission rate in the Cochabamba health facilities.

Department of COCHABAMBA	2004	2005	2006	2007	2008	2009	TOTAL
**Cochabamba City**							
Tested women **(seroprevalence %)**	1551 **(15%)**	3048 **(15%)**	6435 **(14%)**	7347 **(16%)**	11948 **(12%)**	16959 **(13%)**	47288 **(14%)**
Pos./screened children **(Trasm. Rate %)**	6/227 **(3%)**	13/526 **(2%)**	18/527 **(3%)**	26/626 **(4%)**	13/952 **(1%)**	32/1488 **(2%)**	108/4346 **(2%)**
**Punata area**							
Tested women **(seroprevalence %)**	420 **(27%)**	1017 **(25%)**	941 **(22%)**	1303 **(27%)**	1672 **(21%)**	2509 **(19%)**	7862 **(22%)**
Pos./screened children **(Trasm. Rate %)**	4/35 **(11%)**	3/84 **(4%)**	2/120 **(2%)**	3/167 **(2%)**	4/259 **(2%)**	2/183 **(1%)**	18/848 **(2%)**
**Sacaba and Quillacollo area**							
Tested women **(seroprevalence %)**	918 **(15%)**	2140 **(14%)**	5448 **(16%)**	6935 **(15%)**	8674 **(13%)**	10603 **(15%)**	34718 **(15%)**
Pos./screened children **(Trasm. Rate %)**	4/111 **(4%)**	2/274 **(1%)**	16/557 **(3%)**	12/680 **(2%)**	17/653 **(3%)**	21/734 **(3%)**	72/3009 **(2%)**
**South Cone Area**							
Tested women **(seroprevalence %)**				1175 **(67%)**	1382 **(65%)**	1421 **(62%)**	3978 **(65%)**
Pos./screened children **(Trasm. Rate %)**				11/277 **(4%)**	7/449 **(2%)**	17/459 **(4%)**	35/1185 **(3%)**
**Chapare (non endemic area)**							
Tested women **(seroprevalence %)**			3172 **(17%)**	3884 **(15%)**	5466 **(19%)**	6602 **(21%)**	19124 **(19%)**
Pos./screened children **(Trasm. Rate %)**			2/196 **(1%)**	9/293 **(3%)**	19/692 **(3%)**	14/567 **(2%)**	44/1748 **(3%)**
**Totora and Capinota**							
Tested women **(seroprevalence %)**				5059 **(27%)**	6848 **(28%)**	8023 **(28%)**	23102 **(27%)**
Pos./screened children **(Trasm. Rate %)**				20/570 **(4%)**	26/1141 **(2%)**	31/1026 **(3%)**	79/2933 **(3%)**

TOTAL COCHABAMBA	2004	2005	2006	2007	2008	2009	TOTAL
Tested women **(seroprevalence %)**	2889 **(16%)**	6205 **(16%)**	15996 **(16%)**	25703 **(21%)**	35990 **(19%)**	46117 **(19%)**	136072 **(19%)**
Pos./screened children **(Trasm. Rate %)**	14/373 **(4%)**	18/884 **(2%)**	38/1400 **(3%)**	81/2613 **(3%)**	86/4146 **(2%)**	117/4457 **(3%)**	356/14069 **(3%)**

Tested women: number of screening tests made during prenatal controls and at delivery by IHA technique.

seroprevalence %: The seroprevalence was calculated based on positive results by IHA if dilution was ≥1/16.

Positive children: total number of positive children diagnosed at birth, before 6 months and between 6 and 12 months.

Transmission rate was calculated with positive children over children born from positive mother tested at birth.

**Table 4:** Seroprevalence and maternal-fetal transmission rate in the Chuquisaca health facilities.

Department of CHUQUISACA	2004	2005	2006	2007	2008	2009	TOTAL
**City of Sucre**							
Tested women **(seroprevalence %)**	3567 **(37%)**	6916 **(35%)**	6838 **(36%)**	6104 **(33%)**	7155 **(32%)**	8078 **(31%)**	38658 **(34%)**
Pos./screened children **(Trasm. Rate %)**	2/430 **(0,5%)**	34/1451 **(2%)**	48/914 **(5%)**	37/807 **(5%)**	16/1018 **(2%)**	16/1659 **(1%)**	153/6279 **(2%)**
**Tarabuco-Monteagudo Area**							
Tested women **(seroprevalence %)**			1643 **(54%)**	1813 **(57%)**	1785 **(57%)**	1830 **(56%)**	7071 **(56%)**
Pos./screened children **(Trasm. Rate %)**			31/305 **(10%)**	29/596 **(5%)**	21/659 **(3%)**	13/659 **(2%)**	94/2219 **(4%)**
**San Lucas-Camargo Area**							
Tested women **(seroprevalence %)**			686 **(41%)**	853 **(48%)**	766 **(49%)**	773 **(43%)**	3078 **(45%)**
Pos./screened children **(Trasm. Rate %)**			5/131 **(4%)**	15/209 **(7%)**	6/155 **(4%)**	7/156 **(4%)**	33/651 **(5%)**

TOTAL CHUQUISACA	2004	2005	2006	2007	2008	2009	TOTAL
Tested women **(seroprevalence %)**	3567 **(37%)**	6916 **(35%)**	9167 **(39%)**	8770 **(39%)**	9706 **(38%)**	10681 **(36%)**	48807 **(38%)**
Pos./screened children **(Trasm. Rate %)**	2/430 **(0,5%)**	34/1451 **(2%)**	84/1350 **(6%)**	81/1612 **(5%)**	43/1832 **(2%)**	36/2474 **(1%)**	280/9149 **(3%)**

**Table 5:** Seroprevalence and maternal-fetal transmission rate in the Tarija health facilities.

Department of TARIJA	2004	2005	2006	2007	2008	2009	TOTAL
**Tarija and surroundings**							
Tested women **(seroprevalence %)**	2380 **(41%)**	4113 **(39%)**	4420 **(35%)**	4165 **(32%)**	5101 **(34%)**	4705 **(36%)**	24884 **(36%)**
Pos./screened children **(Trasm. Rate %)**	16/639 **(3%)**	35/1091 **(3%)**	22/734 **(3%)**	9/683 **(1%)**	28/1348 **(2%)**	25/1714 **(1%)**	135/6209 **(2%)**
**Chaco Area**							
Tested women **(seroprevalence %)**	995 **(49%)**	1925 **(44%)**	2401 **(46%)**	3120 **(47%)**	3223 **(40%)**	3376 **(42%)**	15040 **(44%)**
Pos./screened children **(Trasm. Rate %)**	8/280 **(3%)**	20/687 **(3%)**	25/823 **(3%)**	28/697 **(4%)**	24/803 **(3%)**	32/871 **(4%)**	137/4161 **(3%)**
**Bermejo (non endemic area)**							
Tested women **(seroprevalence %)**			312 **(28%)**	728 **(28%)**	766 **(27%)**	818 **(28%)**	2624 **(28%)**
Pos./screened children **(Trasm. Rate %)**			3/50 **(6%)**	4/121 **(3%)**	3/131 **(2%)**	0/76 **(0%)**	10/378 **(3%)**

TOTAL TARIJA	2004	2005	2006	2007	2008	2009	TOTAL
Tested women **(seroprevalence %)**	3375 **(43%)**	6038 **(40%)**	7133 **(38%)**	8013 **(37%)**	9090 **(35%)**	8899 **(38%)**	42548 **(38%)**
Pos./screened children **(Trasm. Rate %)**	24/919 **(3%)**	55/1778 **(3%)**	50/1607 **(3%)**	41/1501 **(3%)**	55/2282 **(2%)**	57/2661 **(2%)**	282/10748 **(3%)**

**Table 6:** Seroprevalence and maternal-fetal transmission rate in the Santa Cruz health facilities.

Department of SANTA CRUZ	2007	2008	2009	TOTAL
**Santa Cruz city and surroundings**				
Tested women **(seroprevalence %)**	12976 **(19%)**	26762 **(18%)**	33041 **(20%)**	72779 **(19%)**
Pos./screened children **(Trasm. Rate %)**	7/514 **(1%)**	47/3379 **(1%)**	65/4915 **(1%)**	119/8808 **(1%)**
**Montero-Warnes Area**				
Tested women **(seroprevalence %)**	1886 **(19%)**	4701 **(21%)**	4350 **(25%)**	10937 **(22%)**
Pos./screened children **(Trasm. Rate %)**	9/221 **(4%)**	21/703 **(3%)**	33/1062 **(3%)**	63/1986 **(3%)**
**Camiri**				
Tested women **(seroprevalence %)**	735 **(49%)**	1147 **(45%)**	1142 **(42%)**	3024 **(45%)**
Pos./screened children **(Trasm. Rate %)**	3/121 **(2%)**	3/219 **(1%)**	5/415 **(1%)**	11/755 **(2%)**

TOTAL SANTA CRUZ	2007	2008	2009	TOTAL
Tested women **(seroprevalence %)**	15597 **(20%)**	32610 **(19%)**	38533 **(21%)**	86740 **(20%)**

**Table 7:** Seroprevalence and maternal-fetal transmission rate in the Potosí health facilities.

Department of POTOSI	2007	2008	2009	TOTAL
**Potosí city**				
Tested women **(seroprevalence %)**	1946 **(3%)**	3879 **(5%)**	3237 **(4%)**	9062 **(4%)**
Pos./screened children **(Trasm. Rate %)**	0/34 **(0%)**	0/38 **(0%)**	0/40 **(0%)**	0/112 **(0%)**
**Tupiza and Villazón area**				
Tested women **(seroprevalence %)**	799 **(17%)**	2050 **(20%)**	2173 **(18%)**	5022 **(19%)**
Pos./screened children **(Trasm. Rate %)**	0/33 **(0%)**	1/172 **(0,6%)**	1/114 **(0,8%)**	2/319 **(0,6%)**

TOTAL POTOSI	2007	2008	2009	TOTAL
Tested women **(seroprevalence %)**	2745 **(7%)**	5929 **(10%)**	5410 **(10%)**	14084 **(10%)**
Pos./screened children **(Trasm. Rate %)**	0/67 **(0%)**	1/210 **(0,5%)**	154 **(0,6%)**	431 **(0,5%)**

**Table 8:** Seroprevalence and maternal-fetal transmission rate in the La Paz health facilities.

Department of LA PAZ	2007	2008	2009	TOTAL
**Yungas area (La Paz)**				
Tested women **(seroprevalence %)**	693 **(6%)**	2638 **(4%)**	2520 **(5%)**	5851 **(5%)**
Pos./screened children **(Trasm. Rate %)**	0/8 **(0%)**	3/40 **(8%)**	15/47 **(15%)**	10/95 **(11%)**

From the 1,093 children diagnosed, 78% began the treatment (851 children) and 70% completed the 30-day treatment (771 children). From the total of children treated, 122 underwent a first serological control test 6 months post-treatment, and 96% of these came out negative (118/122). Unfortunately, the 4 children without negative serology at 6 months didn't have another serological test later, so we have no ways of knowing their final outcome. No child has received rescue treatment.

As for the group that did not begin the treatment (242 children, 22%), the main reason was that mothers had already left the maternity unit before getting to know the test result. Five reported cases were not treated because the parents rejected the treatment. Six reported cases, mainly newborns with low weight, were not treated because they manifested another concomitant pathology, and therefore the neonatologist considered that the use of Benznidazole along with another medication was contraindicated in those newborns. Afterwards, the follow up on these children was disrupted or they in fact died (hepatitis, toxoplasmosis, severe cardiopathy, intestinal malformation, etc.).

Within the 10% of children who began the treatment and later abandoned it (80 cases), the main reasons evoked were: difficulty for the parents to attend to the weekly checkups at that facility, changes of address, travel to another department, difficulties in the reference to a lower level facility which was not in conditions to monitor the treatment, and death during the treatment (15 deaths reported due to reasons unrelated to the treatment).

## Discussion

This work constitutes certainly, the most important work in congenital Chagas disease in Bolivia, and could serve as a solid basis for future studies or as baseline for future implementations in congenital Chagas disease programs. We show, for the first time, that it is possible to implement a program for diagnosis and treatment of congenital Chagas in all areas of Bolivia – both urban and rural. The minimum conditions required for these implementations are: a health facility with a laboratory and resources available for activities of training, recycling, evaluation, monitoring, and, most important, very motivated health personnel. Unfortunately, however, the people that live in dispersed rural areas of the 164 endemic municipalities, which represent around 37% of the national population [41] still remain out of reach. According to the 2001 population data, the degree of urbanization in Bolivia is 62.4%. From this urban population, 71.9% live in main cities, and 10.2% live in middle size towns. These people only have access to level 1 facilities, where they are generally attended by a nurse-aid, without a laboratory facility.

The implemented strategy has been adapted to the context of Bolivia taking into consideration certain characteristics of the health system and the population. The first adjustment was to carry out the serology in children born from positive mothers after 6 months in order to make the Chagas control coincide with the third doses of pentavalent vaccine, instead of performing it at 8–9 months; this was done because it has been seen a significantly larger percentage of children going into the health facilities after 6 months as opposed to the recommended 8–9 months of age. The second adjustment was the micromethod control between 1 and 6 months of age, preferably at 2 months, and at the same time as the first dose of the pentavalent vaccine; this control was based on the observation of a tendency to low parasitemia detection at birth, which can be easier to detect one or two months later, as recommended by Zaidemberg et al [17] and by the OPS following the International Conference for congenital Chagas in Cochabamba [42].

During the first three years of work (2004–2006) we have verified, at various reference hospitals (Hospital San Juan de Dios in Tarija, Gineco-obstetric Hospital in Sucre and German Urquidi Maternity Unit in Cochabamba), that the detection with positive micromethods performing only one micromethod control at birth was lower than expected, and by introducing a second micromethod control in the beginning of 2006, 21% of the congenital Chagas cases were detected. The benefit of this change in strategy is an earlier diagnosis and consequently earlier treatment of the infection, which, when treated in its acute phase, favors a quicker recuperation, and shortens the time of exposure to the parasite [33].

The seroprevalence results obtained from pregnant women reflect the prevalence of Chagas disease among the adult population of both sexes in the same age group (between 14 and 50) – if we consider that gender equality exists in the exposure to the vector. We can observe hyper-endemic hotspots where prevalence surpasses 60%: 65% in the peri-urban areas of Tarija, 66% in the municipality of Aiquile of Cochabamba, and 78% in the municipality of Tomina, Chuquisaca.

Until the beginning of the program in 2004, some studies were carried out in Bolivia, generally limited to localized results making it difficult to compare them with those of the current program. In Cochabamba, in two different studies from 1992 to 2001, the seroprevalence dropped from 27.6% to 17.7%. Following the some tendency now, the seroprevalence is being set around 13%. In Santa Cruz, between November 2006 and June 2007, in the Japanese University Hospital, Bern et al. detected a seroprevalence among pregnant women of 29% and the congenital program achieved a seroprevalence among pregnant women of 23.7%. In the Percy Boland hospital of Santa Cruz, in 1980, Azogue et al. attained a 51% seroprevalence in pregnant women [25]; in the 2008–2009 period, the program achieved a 19.6% seroprevalence, showing a significant drop in seroprevalence in the population that attended this health facility. In the Chaco area (south of Bolivia) in a study carried out by Chippaux et al. in Carapari (Tarija) [43], the seroprevalence found in the general population was 51.2%; in 2008, the same program calculated a 46.6% seroprevalence among pregnant woman. In Yacuiba, a city near Carapari, during the period of May 2003 to November 2004, Salas et al. [44] established 42.2% prevalence among pregnant women; during 2005 the program achieved a similar prevalence of 41.7%. The differences in seroprevalence in pregnant women are above all, related to the area where the patients have lived most of their life; In Areas were the infestation of the vector were higher, the seroprevalence of Chagas disease was also higher.

In this work, it has been established that the maternal-fetal transmission rate obtained has an average of 2.6%, fluctuating between 1.4% in cases detected at birth, 2.3% in cases detected with the 2^nd^ micromethod control before 6 months, and 3.3% with the serology control between 6 and 12 months of age. This result differs from the study carried out in German Urquidi Maternity unit during the periods of 1992–1994 and 1999–2001, where a 4.9% and 5.9% transmission rate was achieved, respectively [28]. That rate is also connected to the facilities' detection capabilities. By evaluating the facilities separately we have noticed that differences are related to compliance with the norm (3 control tests) and to the determining capacity a facility has: Quality of laboratory equipment, technical capacity and motivation of the personnel in charge of micromethod readings, team work between medical, nursing and laboratory personnel, and job stability of the qualified personnel.

We have observed differences in the maternal-fetal transmission rate among the health facilities, or even at the same facility as years pass. This fact coincides with other studies where fluctuations can be seen in the maternal-fetal transmission rate at a specific health facility [45] and variations within the same country and between countries [18], [46]. However, in the health facilities where the transmission rate was found to be higher than 10%, or having more radical fluctuations, technical supervisions were carried out with the objective of detecting possible flaws in the technical procedures, interpretation of results, or compliance with control tests.

At first we began considering a 5% transmission rate, based on previous studies carried out in Bolivia [28], but the transmission rate we found has been 2.6%. In an urban area of the city of Buenos Aires, De Rissio et al [45] found a 6.1% average transmission rate using micromethod between birth to 6 months of age, and micromethod plus serology between 6 and 12 months of age. In De Rissio's work, 68.9% of the cases were detected using the micromethod before 6 months of age. We found, employing a similar strategy in two parasitological control tests, 76% using the micromethod technique. In Santa Cruz (Bolivia), C Bern et al. [47] used micromethod and PCR for diagnostic of congenital Chagas disease and found differences between both methods; 2.6% of transmission rate using micromethod compared with 3.8% using the PCR technique.

The difference in the global rate of transmission between De Risio's study and the results in Bolivia, can be explained by the fact that in De Risio's study there is a follow up to one year of age on 44.2% of the children born from positive mothers, while during the implementation of the program in Bolivia only 18% of the children born from positive mothers have returned for their 2^nd^control between 6 and 12 months of age. The follow up with the three control methods until 1 year of age is the key in detecting the majority of cases.

Following the example of De Rissio's work, we could consider fitting two techniques into each test carried out on the child (parasitological and serological) in order to increase diagnosis probabilities, but this would significantly increase workload at the laboratory.

Another factor that affects the detection of cases is the probable low parasitemia at birth, due to the lower probability of re-infection during pregnancy – which is thanks to the effectiveness of vector control, mainly in big urban areas [28].

In order to know the real transmission rate, a broad pilot study with a strict follow up should be carried out on a selected group of children born from positive mothers, throughout a whole year, and at different locations of the country where it can be guaranteed there is no risk of other means of transmission.

The current program has managed to detect around 50% of the expected cases. Therefore, it is even more important to consolidate the consecutive control tests up to 1 year of age and include level 1 facilities into the program. These considerations must be taken into account by the current authorities in the National Program and in local health organizations.

Most of the 1,093 congenital cases detected were diagnosed in level 2 and 3 facilities, which confirms the strategy's acceptance by health professionals. In spite of various internal organization problems, progress can be seen in the program's integration at such level. However, it is necessary to include level 1 facilities into the program in order to improve the monitoring capacity of children born from positive mothers and increase detection of cases from 6 months of age and older.

The treatment carried out in 70% of diagnosed children is a great achievement for the Bolivian health system, considering that only local resources were employed. The achievement is also due to the Chagas law of Mars 23^rd.^ 2006, which declares that the prevention and fight against Chagas disease is a national priority. The mentioned law facilitated the assimilation of the program as a professional duty. Other programs, such as the national tuberculosis program, which counts with more funds and 14 more years of implementation [48], [49], currently manages 81% treatment coverage (Situación de Salud en Bolivia 2004. Ministerio de Salud y Deportes). This fact leads us to speculate the probable need of many more years of work for the consolidation period of the congenital Chagas program in Bolivia.

In more than 90% of the treated cases, no adverse effects were seen. The adverse effects were adequately treated by the pediatricians and general doctors. None of them justifies a definite withdrawal from the treatment. This fact demonstrates the safety and the lack of side effects in babies treated with BZN, compared with treatment in adults, in whom the percentages of side effects can rise between 30% and 80% [50], [51]. Recently, a new pediatric formulation of 12.5 mg has been fabricated; once available in Bolivia, the treatment will be easier to give to children.

On the other hand, in spite of the efforts from the health personnel, it is necessary to point out that 8% of the children were unable to complete the treatment: this was due, to a great extent, to faults in the reference system, and problems in the accessibility to health facilities were treatment was begun.

Another problem concerning treatment is the time that mothers remain at the maternity unit during the postpartum period. In general the stay is supposed to be 24 hrs., but it comes to be less than 12 hrs. in very saturated maternity units. For this reason it is not possible to obtain the test results on time. After their departure from the maternity unit it is difficult to locate the mothers to advise them regarding the monitoring or treatment of their baby, often because there is no personnel available to look for them, and in many cases because the home details are ambiguous or incomplete.

The serological negativization rate found was a little lower than that found in other articles, 98% vs. 100%. Various articles show that the cure rate for congenital Chagas when treatment starts before 12 months of age is 100% [30]. Our observations suggest that in order to certify this, a study would have to be carried out on a broad group of children treated with quantified serology tests performed until negativization is achieved. However, taking into account the children's low return rate to control testing, and the cure rate being close to 100%, we could omit post-treatment control and only carry it out in cases with suspicion of failure to comply with the medication, or when mother and child live in areas with vector presence, or in sentinel groups. That said, other authors recommend post treatment control starting at 10 months of age in children that were treated at birth [52].

Another benefit brought by the work carried out has been the strengthening of the national system of epidemiological surveillance information. This system is in charge of providing the country and the health sector with details and information for epidemiological surveillance and management. While in 2004 the only existent indicators in the national forms referred to vector control, currently there are also indicators for diagnoses and treatment which must be sent on a monthly basis by all facilities that work with the congenital Chagas program. The gathered information has been used to create coverage indicators and to set program targets (see point 3 in methods).

An important induced effect is the demand for treatment from the infected mothers. Even though there is no program established for adult treatment, various health facilities have started to treat women starting from 6 months post-partum, once the child is no longer on exclusive breastfeeding. When treating women in fertile age, as well as the beneficial effect of treating the person, the risk of maternal-fetal transmission may be reduced. However, only a recent study performed in Spain [53] show the reduction of congenital transmission in women treated with Benznidazole and remains an area that still needs to be investigated.

It's important to summarize some of the limitations of the program. By using IHA instead of ELISA in the pregnant women screening, probably we lost around 5 to 10% of positive mothers, but the IHA was the only test available in routine laboratories. The lack of follow up of children born from a positive woman is probably the most important problem; If it had been possible to perform three control tests to the 42,538 children born from positive mothers during that period, 1,276 expected cases would have been detected with a maternal-fetal transmission rate of 3% or 2,127 cases with a 5% transmission rate, instead of the 1,093 cases detected. It's necessary to have a budget for continuous information campaigns and monitoring activities at the health facilities. Diagnosis, treatment, and follow up of congenital Chagas disease are included in the routine protocols of the Ministry of Health so, there is no cost for the population and the program is sustainable over the time, but continuous monitoring activities are needed to keep the good quality of the program and to maintain the motivation of the heath personnel. Recently, Sicuri et al showed that financially, the best Chagas strategy for non-endemic countries is Chagas screening of all Latin American women and their infants. Considering its low cost, it follows also that it is the best alternative for endemic countries as well [54].

### Conclusion

Congenital Chagas disease is a recognized public health issue and must be included in the priorities of the countries with infected population. More awareness is needed on behalf of authorities in order to fight Chagas disease. It is also essential to continue studying the strategies which are most applicable to rural contexts in order to begin detection and treatment in less accessible and marginalized populations.

We also recommend, as other authors, [19], [46], that special efforts must be made to achieve the detection of congenital Chagas in families with mothers who have Chagas, more so if a case of congenital Chagas has been proven within the family.

In conclusion, it has been shown that it is possible, to implement a National Congenital Chagas Program following the PAHO recommendations of early detection and treatment of cases under 1 year old with limited resources. Yet it is necessary to continue the supervision and training activities in order to maintain the interest of health professionals, increase current coverage, and improve the overall quality of the program.

## References

[1] WHO (2002) WHO Expert Committee on the Control of Chagas Disease (2000: Brasilia, Brazil). Data WLC-i-P, editor. Control of Chagas disease : second report of the WHO expert committee. 1–109.

[2] Pan American Health Organization, WHO Program on Neglected Tropical Diseases (2007) Estimación cuantitativa de la enfermedad de Chagas en las Américas [In Spanish]. Washington, Pan American Health Organization. (OPS/HDM/CD/425-06)

[3] Schmunis GA, Yadon ZE (2010) Chagas disease: a Latin American health problem becoming a world health problem. Acta Tropica 115: 14–21. 19932071 10.1016/j.actatropica.2009.11.003

[4] Lucas RM, Barba MC (2009) Prevalence of american trypanosomiasis in pregnant women from a health area of Valencia, Spain: 2005–2007. Revista Espanola de Salud Pública 83: 543–555. 19893882

[5] Dejour Salamanca D, La Ruche G, Tarantola A, Degail MA, Jeannel D, et al (2009) Chagas disease in France: estimated number of infected persons and cardiac diseases in 2009, by risk groups. Bulletin de la Societe de Pathologie Exotique 102: 285–290. 20131421

[6] Gonzalez-Granado LI, Rojo-Conejo P, Ruiz-Contreras J, Gonzalez-Tome MI (2009) Chagas disease travels to Europe. Lancet 373: 2025. 10.1016/S0140-6736(09)61110-7 19524777

[7] Schmunis GA (2007) Epidemiology of Chagas disease in non-endemic countries: the role of international migration. Memorias do Instituto Oswaldo Cruz 102 Suppl 1: 75–85. 17891282 10.1590/s0074-02762007005000093

[8] Freilij H AJ, Storino R, (1994) Chagas congéinto. In: Storino R MJ, editor. Enfermedad de Chagas. Buenos Aires: Doyma. pp. 267–278.

[9] Gomes YM, Lorena VM, Luquetti AO (2009) Diagnosis of Chagas disease: what has been achieved? What remains to be done with regard to diagnosis and follow up studies? Memorias do Instituto Oswaldo Cruz 104 Suppl 1: 115–121. 19753466 10.1590/s0074-02762009000900017

[10] Martinez de Tejada B, Jackson Y, Paccolat C, Irion O (2009) Congenital Chagas disease in Geneva: diagnostic and clinical aspects. Revue Medicale Suisse 5: 2091–2092, 2094–2096. 19947451

[11] Brutus L, Santalla JA, Salas NA, Schneider D, Chippaux JP (2009) Screening for congenital infection by *Trypanosoma cruzi* in France. Bulletin de la Societe de Pathologie Exotique 102: 300–309. 20131424

[12] Chagas disease: a neglected emergency. (2009) The Lancet,Vol 373, DOI: 10.1016/S0140-6736 (09)61002-3. 10.1016/S0140-6736(09)61002-3 19482198

[13] Flores-Chavez M, Faez Y, Olalla JM, Cruz I, Garate T, et al (2008) Fatal congenital Chagas' disease in a non-endemic area: a case report. Cases Journal 1: 302. 18992159 10.1186/1757-1626-1-302 PMC2585580

[14] Neto EC, Rubin R, Schulte J, Giugliani R (2004) Newborn screening for congenital infectious diseases. Emerging Infectious Diseases 10: 1068–1073. 15207059 10.3201/eid1006.030830 PMC3323166

[15] Jackson Y, Myers C, Diana A, Marti HP, Wolff H, et al (2009) Congenital transmission of Chagas disease in Latin American immigrants in Switzerland. Emerging Infectious Diseases 15: 601–603. 19331743 10.3201/eid1504.080438 PMC2671437

[16] Luquetti AO, Dias JC, Prata A (2005) Diagnosis and treatment of congenital infection caused by *Trypanosoma cruzi* in Brazil. Revista da Sociedade Brasileira de Medicina Tropical 38 Suppl 2: 27–28. 16482809

[17] Zaidenberg M (1999) Congenital Chagas' disease in the province of Salta, Argentina, from 1980 to 1997. Revista da Sociedade Brasileira de Medicina Tropical 32: 689–695. 10881107 10.1590/s0037-86821999000600012

[18] Sosa-Estani S (2005) Congenital transmission of *Trypanosoma cruzi* infection in Argentina. Revista da Sociedade Brasileira de Medicina Tropical 38 Suppl 2: 29–32. 16482810

[19] Sanchez Negrette O, Mora MC, Basombrio MA (2005) High prevalence of congenital *Trypanosoma cruzi* infection and family clustering in Salta, Argentina. Pediatrics 115: e668–672. 15930194 10.1542/peds.2004-1732

[20] Blanco SB, Segura EL, Cura EN, Chuit R, Tulian L, et al (2000) Congenital transmission of *Trypanosoma cruzi*: an operational outline for detecting and treating infected infants in north-western Argentina. Tropical Medicine & International Health 5: 293–301. 10810029 10.1046/j.1365-3156.2000.00548.x

[21] de Andrade AL, Zicker F, Martelli CM (1994) An epidemiological approach to study congenital Chagas' disease. Cadernos de saude publica/Ministerio da Saude, Fundacao Oswaldo Cruz, Escola Nacional de Saude Publica 10 Suppl 2: 345–351. 10.1590/s0102-311x1994000800012 15042224

[22] Schenone H, Gaggero M, Sapunar J, Contreras MC, Rojas A (2001) Congenital Chagas disease of second generation in Santiago, Chile. Report of two cases. Revista do Instituto de Medicina Tropical de Sao Paulo 43: 231–232. 11558005 10.1590/s0036-46652001000400011

[23] Cusnaider CM GRD, Amat L, Aguiló F, Hernández A, Lailla JM (2004) Chagas congénito ¿Es posible en España? Ginecología y Obstertricia Clínica. pp. 198–203. Available: http://www.nexusediciones.com/pdf/gine2004_4/gi-5-4-003.pdf

[24] Munoz J, Coll O, Juncosa T, Verges M, del Pino M, et al (2009) Prevalence and vertical transmission of *Trypanosoma cruzi* infection among pregnant Latin American women attending 2 maternity clinics in Barcelona, Spain. Clinical Infectious Diseases 48: 1736–1740. 19438393 10.1086/599223

[25] Azogue E, La Fuente C, Darras Ch (1981) Transmisión Congénita de la Enfermedad de Chagas en Santa Cruz-Bolivia-I-Epidemiología. Bol Inf CENETROP II: 23–29.

[26] Azogue E, La Fuente C, Darras C (1985) Congenital Chagas' disease in Bolivia: epidemiological aspects and pathological findings. Transactions of the Royal Society of Tropical Medicine and Hygiene 79: 176–180. 3923667 10.1016/0035-9203(85)90328-1

[27] Azogue E, Darras C (1995) Congenital Chagas in Bolivia: comparative study of the effectiveness and cost of diagnostic methods. Revista da Sociedade Brasileira de Medicina Tropical 28: 39–43. 10.1590/s0037-86821995000100007 7724866

[28] Torrico F, Alonso-Vega C, Suarez E, Rodriguez P, Torrico MC, et al (2004) Maternal *Trypanosoma cruzi* infection, pregnancy outcome, morbidity, and mortality of congenitally infected and non-infected newborns in Bolivia. The American Journal of Tropical Medicine and Hygiene 70: 201–209. 14993634

[29] Chagas en Bolivia. El trabajo del Programa Piloto de Control de Chagas SNS/CCH. (1994) Agencia Internacional de Desarrollo de los EEUU en Bolivia, P.L.480

[30] Congenital infection with *Trypanosoma cruzi*: from mechanisms of transmission to strategies for diagnosis and control. Revista da Sociedade Brasileira de Medicina Tropical 36: 767–771. 15143784 10.1590/s0037-86822003000600024

[31] Carlier Y, Torrico F (2005) Coloquio Internacional Infección congénita por *Trypanosoma cruzi*:desde los mecanismos de transmisión hasta una estrategia de diagnóstico y control. Revista da Sociedade Brasileira de Medicina Tropical 38: 114–118.

[32] PAHO (2004) PAHO Consultation on Congenital Chagas Disease, Its Epidemiology and Management OPS/DPC/CD/301/04.

[33] Torrico F, Alonso-Vega C, Suarez E, Billot C, Torrico M, et al.. (2007) Chagas congénito: Estrategias de Diagnóstico y Control. Ministerio de Salud y Deportes PNdC, editor. Cochabamba, Bolivia.

[34] Feilij H, Muller L, Gonzalez Cappa SM (1983) Direct micromethod for diagnosis of acute and congenital Chagas' disease. Journal of Clinical Microbiology 18: 327–330. 6413530 10.1128/jcm.18.2.327-330.1983 PMC270800

[35] Woo PT (1971) Evaluation of the haematocrit centrifuge and other techniques for the field diagnosis of human trypanosomiasis and filariasis. Acta Tropica 28: 298–303. 4400769

[36] Torrico MC, Solano M, Guzman JM, Parrado R, Suarez E, et al (2005) Estimation of the parasitemia in *Trypanosoma cruzi* human infection: high parasitemias are associated with severe and fatal congenital Chagas disease. Revista da Sociedade Brasileira de Medicina Tropical 38 Suppl 2: 58–61. 16482816

[37] Suarez E, Alonso-Vega C, Torrico F, Cordova M (2005) Integral treatment of congenital Chagas disease: the Bolivian experience. Revista da Sociedade Brasileira de Medicina Tropical 38 Suppl 2: 21–23. 16482807

[38] el Bouhdidi A, Truyens C, Rivera MT, Bazin H, Carlier Y (1994) *Trypanosoma cruzi* infection in mice induces a polyisotypic hypergammaglobulinaemia and parasite-specific response involving high IgG2a concentrations and highly avid IgG1 antibodies. Parasite Immunology 16: 69–76. 8015857 10.1111/j.1365-3024.1994.tb00325.x

[39] Fescina RHBB, De Mucio B, Martínez G, Díaz Rossello JL, Camacho V, Simini F, Mainero L (2007) Sistema informático perinatal. Historia Clínica perinatal; Instrucciones de llenado y definición de términos. Montevideo

[40] Rojas Cortez M, Avalos M, Rocha V, Goria D. (2007) Distribución geográfica de los Triatominos en Bolivia: discriminación de la distribución de las especies en relación a variables ambientales. In: Ministerio de Salud y Deportes PNdC, editor. Triatominos de Bolivia y la enfermedad de Chagas. Bolivia. pp. 67–139.

[41] Ministerio de Salud y Deportes, Dirección de Planificación y Cooperación externa (2006) Situación de Salud en Bolivia 2004.Documentos de Divulgación Científica

[42] Torrico F, Alonso-Vega C, Billot C, Truyens C, Carlier Y (2007) Relaciones materno-fetales en la infección con *T. cruzi* y la implementación de un programa nacional de detección y tratamiento de Chagas congénito en Bolivia. Enfermedades Emergentes 9: 9–16.

[43] Chippaux JP, Postigo JR, Santalla JA, Schneider D, Brutus L (2008) Epidemiological evaluation of Chagas disease in a rural area of southern Bolivia. Transactions of the Royal Society of Tropical Medicine and Hygiene 102: 578–584. 18430443 10.1016/j.trstmh.2008.03.008

[44] Salas NA, Cot M, Schneider D, Mendoza B, Santalla JA, et al (2007) Risk factors and consequences of congenital Chagas disease in Yacuiba, south Bolivia. Tropical Medicine & International Health 12: 1498–1505. 18076558 10.1111/j.1365-3156.2007.01958.x

[45] De Rissio AM, Riarte AR, Garcia MM, Esteva MI, Quaglino M, et al (2010) Congenital *Trypanosoma cruzi* infection. Efficacy of its monitoring in an urban reference health center in a non-endemic area of Argentina. The American Journal of Tropical Medicine and Hygiene 82: 838–845. 20439964 10.4269/ajtmh.2010.08-0383 PMC2861371

[46] Schijman A (2007) Congenital Chagas Disease In: Mushahwar IK, editor. Congenital and Other Related Infectious Diseases of the Newborn: Elsevier B.V. pp. 223–258.

[47] Bern C, Verastegui M, Gilman RH, Lafuente C, Galdos-Cardenas G, et al (2009) Congenital *Trypanosoma cruzi* transmission in Santa Cruz, Bolivia. Clinical Infectious Diseases 49: 1667–1674. 19877966 10.1086/648070 PMC5454522

[48] Ferrel M (1996) Evolución Epidemiológica de la Tuberculosis en Bolivia. Archivos Bolivianos de Historia de la Medicina 2: 125–129.

[49] Ministerio de Salud y Deporte (2008) Anuario 2008. Linea de base de la Estrategia Integral e Intersectorial de Prevención y Control de la Enfermedad de Chagas en Bolivia 2008–2015. La Paz: Ministerio de Salud y Deporte. Programa Nacional de Chagas.

[50] Pinazo MJ, Guerrero L, Posada E, Rodriguez E, Soy D, et al (2013) Benznidazole-related adverse drug reactions and their relationship to serum drug concentrations in patients with chronic chagas disease. Antimicrobial Agents and Chemotherapy 57: 390–395. 23114763 10.1128/AAC.01401-12 PMC3535922

[51] Viotti R, Vigliano C, Lococo B, Alvarez MG, Petti M, et al (2009) Side effects of benznidazole as treatment in chronic Chagas disease: fears and realities. Expert Review of Anti-infective Therapy 7: 157–163. 19254164 10.1586/14787210.7.2.157

[52] Chippaux JP, Clavijo AN, Santalla JA, Postigo JR, Schneider D, et al (2010) Antibody drop in newborns congenitally infected by *Trypanosoma cruzi* treated with benznidazole. Tropical Medicine & International Health 15: 87–93. 19968839 10.1111/j.1365-3156.2009.02431.x

[53] Murcia L, Carrilero B, Munoz-Davila MJ, Thomas MC, Lopez MC, et al (2013) Risk factors and primary prevention of congenital chagas disease in a nonendemic country. Clinical Infectious Diseases 56: 496–502. 23097582 10.1093/cid/cis910

[54] Sicuri E, Munoz J, Pinazo MJ, Posada E, Sanchez J, et al (2011) Economic evaluation of Chagas disease screening of pregnant Latin American women and of their infants in a non endemic area. Acta Tropica 118: 110–117. 21396345 10.1016/j.actatropica.2011.02.012

